# Orthrus: Towards Evolutionary and Functional RNA Foundation Models

**DOI:** 10.1101/2024.10.10.617658

**Published:** 2024-10-12

**Authors:** Philip Fradkin, Ruian Shi, Keren Isaev, Brendan Frey, Quaid Morris, Leo J. Lee, Bo Wang

**Affiliations:** 1 Vector Institute, Ontario, Canada; 2 Computer Science, University of Toronto, Ontario, Canada; 3 Computational and Systems Biology Program, Sloan Kettering Institute, New York, United States; 4 New York Genome Center, New York, United States; 5 Systems Biology, Columbia University, New York, United States; 6 Electrical and Computer Engineering, University of Toronto, Ontario, Canada; 7 Peter Munk Cardiac Center, University Health Network, Ontario, Canada

**Keywords:** Contrastive Learning, Genomics, Self-Supervised Learning, RNA, Orthology, Representation Learning, Alternative Splicing

## Abstract

In the face of rapidly accumulating genomic data, our understanding of the RNA regulatory code remains incomplete. Pre-trained genomic foundation models offer an avenue to adapt learned RNA representations to biological prediction tasks. However, existing genomic foundation models are trained using strategies borrowed from textual or visual domains, such as masked language modelling or next token prediction, that do not leverage biological domain knowledge. Here, we introduce Orthrus, a Mamba-based RNA foundation model pre-trained using a novel self-supervised contrastive learning objective with biological augmentations. Orthrus is trained by maximizing embedding similarity between curated pairs of RNA transcripts, where pairs are formed from splice isoforms of 10 model organisms and transcripts from orthologous genes in 400+ mammalian species from the Zoonomia Project. This training objective results in a latent representation that clusters RNA sequences with functional and evolutionary similarities. We find that the generalized mature RNA isoform representations learned by Orthrus significantly outperform existing genomic foundation models on five mRNA property prediction tasks, and requires only a fraction of fine-tuning data to do so.

Mature RNAs, resulting from transcription and alternative splicing of precursor RNAs, encode essential genetic information for protein synthesis. The regulation of precursor RNAs is often tightly linked to their sequence and is critical in modulating protein expression and cellular functions [1]. Experimental procedures such as eCLIP, ribosome profiling, or SLAM seq have been pivotal in studying these RNA regulatory processes, but these techniques are often time-consuming and expensive [2–4]. As an alternative, supervised machine learning models trained on genetic sequences can learn the mechanisms of RNA regulation directly from data, thus offering effective and low-cost prediction of cellular processes such as alternative splicing and RNA degradation [5–7]. These models can be used to identify disease mechanisms [8, 9], improve therapeutics such as messenger RNA (mRNA) vaccines [10], and predict the effects of perturbations [11]. Despite the importance of these applications, the difficulty associated with experimental acquisition of training data restricts the use of supervised methods for a wider range of tasks.

Several recent works [12–14] have proposed foundation models as an alternative for supervised learning approaches in genomic domains. Genomic foundation models use deep neural networks to learn an expressive representation of genetic sequences by pre-training on large datasets. During pre-training, self-supervised learning (SSL) objectives are used to train the model in the absence of labeled examples. SSL can be formulated through a data reconstruction objective, where a model is required to reconstruct a portion of the input data. Existing genomic foundation models use training objectives including next token prediction (NTP) and masked language modeling (MLM) [12, 15, 16]. Foundational models that effectively capture the underlying biological complexities enable few-shot learning, generalizing experimental biology using a minimal number of samples [17, 18]. Representations learned with foundation model techniques can be fine-tuned on related downstream tasks with fewer labeled data points, reducing reliance on data collection and demonstrating impressive generalization capabilities to a diversity of tasks [19, 20]. However, the unique properties inherent to genomic data pose challenges for implementing reconstruction-based SSL objectives or supervised learning approaches.

Genomic sequences in the natural world are constrained by evolutionary viability, resulting in low natural diversity^1^ and high mutual information across genomes from the same species [22]. Latest estimates propose that approximately ten percent of the human genome is under constraint and can be considered high information content [23, 24]. The remaining 90% of the genetic sequence lacks evidence of negative selection, meaning mutations may have little to no impact on organism fitness [25, 26]. Without a strong biological inductive bias, existing reconstruction-based SSL models often reconstruct non-informative tokens, which can result in sub-optimal representations. Due to the high-mutual information between samples, it is also difficult to scale the effective size of the training dataset to circumvent this issue. As we later show, applications of SSL methods to genomics learn latent representations that are not well suited for RNA property prediction [12–14, 27]. The gap between baseline SSL methods and supervised approaches remains large, while no clear trend exists between model size and performance.

Here, we propose Orthrus, an RNA foundation model that is pre-trained on mature RNA sequences. Orthrus uses a novel biologically motivated contrastive learning objective to structure the model latent space by maximizing similarity between splicing isoforms and evolutionary related transcripts [28, 29]. Using this contrastive objective, Orthrus is pre-trained on splicing annotation data from 10 species and orthologous alignments from more than 400 mammalian species in Project Zoonomia [30]. Pre-training Orthrus on mature RNAs with high functional importance and sequence conservation further allows Orthrus to focus on sequence regions with high information content [23, 25]. Orthrus is trained using a Mamba encoder, which enables favorable model properties such as the learning of variable motif spacing, context filtration, and linear memory scaling with sequence length [31]. Orthrus pre-training results in effective mature RNA representations that are predictive of diverse RNA properties.

We show that Orthrus’s learned representations can be used to accurately predict the properties of mature mammalian RNA sequences in three key contexts. First, we test the effectiveness of biologically inspired contrastive learning by fitting a linear model on top of the pre-trained latent representations. We identify that Orthrus outperforms other self-supervised foundation models, and applying this simple linear transformation approaches the performance of supervised methods on all property prediction tasks. Second, we fine-tune the pre-trained models on experimentally collected RNA property datasets and demonstrate state-of-the-art performance when generalizing to unseen sequences. Orthrus is able to effectively perform in the low data regime, requiring as few as 45 labeled examples to fine-tune an RNA half-life predictor. Finally, we identify that increasing the model size improves performance, opening up the door for further improvements by scaling both the training dataset and model size.

## 2 Results

### Contrastive Learning Dataset Construction

Orthrus is trained using contrastive learning, which constructs a structured representation space by directly maximizing the embedding similarity within positive pairs of related RNA transcripts while minimizing the similarity of all unrelated transcripts. Each positive contrastive pair consists of a reference RNA transcript that is paired with a transcript sampled from a set of related transcripts. Under the contrastive learning framework, these related transcripts can be viewed as augmentations of the reference transcript, as they share similarities in function and property but differ in sequence. We construct a contrastive learning dataset that identifies positive pairs based on alternative splicing and orthologous transcripts produced through mammalian speciation events. Our hypothesis is that identified positive pairs of mRNA sequences produced from these processes are more functionally similar to one another than a randomly sampled RNA sequences.

**Fig. 1:**
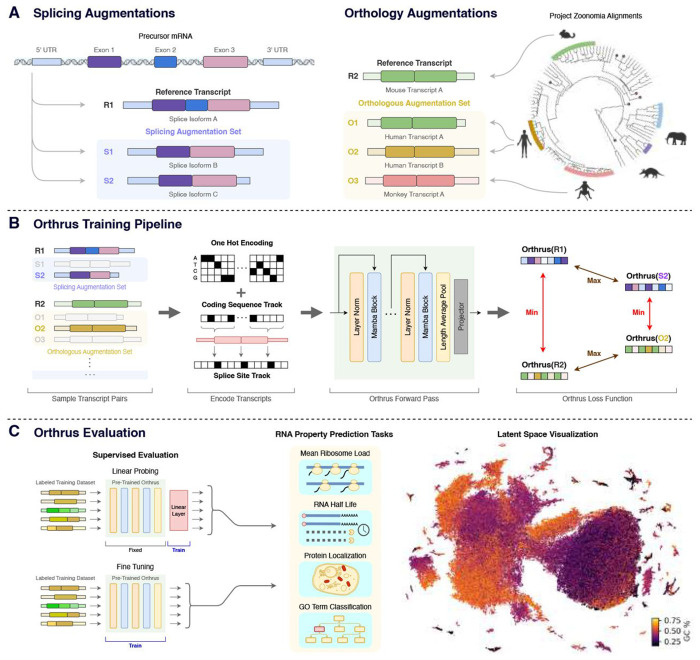
Description of Orthrus dataset construction, training, and evaluation procedures. (**A**) Contrastive dataset construction. We treat each RNA transcript in the pre-training dataset as a reference transcript. For each reference transcript, we identify sets of transcripts that are related through alternative splicing and orthology processes. These related transcripts are viewed as augmentations of the reference transcript under the contrastive learning framework. Each reference transcript can be associated to both splicing and orthology augmentation sets. (**B**) The Orthrus training pipeline. For all reference transcripts in a batch, we randomly sample a positive paired transcript from its splicing and orthology augmentation sets. All transcripts are converted into a six track encoding. We then generate a projection of the sequences using Orthrus model and apply the contrastive loss over these samples, maximizing similarity between positive pairs while minimizing it for all the other transcripts. (**C**) Orthrus evaluation consisting of linear probing, fine-tuning over a variety of mature RNA properties and visualizing the model latent space.

To construct positive pairs based on alternative splicing, we group alternatively spliced transcripts using GENCODE and RefSeq databases depending on availability [32, 33]. We utilize splice information across 10 species, covering a broad range across the evolutionary tree: human, mouse, chicken, C. elegans, chimpanzee, cow, dog, drosophila, rat, and zebrafish. In addition, we make use of naive orthology for positive pair generation: for cases where gene names are consistent across species, we pool the transcripts generated by alternative splicing into the same transcript set (Figure 1 A). Alternatively spliced mRNA isoforms exhibit variability in UTR and coding sequences composition, at times demonstrating novel function. However, our work is based on the assumption that on average splice isoforms are more functionally similar to one another than a randomly sampled mRNA transcripts. We empirically find that sequence diversity present in alternatively spliced isoforms is an effective source of function preserving variation.

Orthologous transcripts from mammalian species present another source of sequence diversity, generated by genetic drift post speciation events [34, 35]. We utilize positive pairs generated through the process of speciation across the Eutheria clade through the Zoonomia TOGA resource, which performs joint gene annotation and orthology inference mapping transcripts from over 400 transcripts to human and mouse annotations [30]. To identify orthologous pairs, TOGA performs alignment over identified coding sequences and neighboring intronic and intergenic regions. We hypothesize that using orthologous sequencing as positive pairs in our dataset can allow the model to learn mRNA regions that are conserved and preserved over evolutionary time due to negative selection. These regions in turn are likely to be functionally important, and relevant for mRNA property prediction.

Overall, our final dataset contains 49 million unique transcripts and over 870 million unique positive pairs (Table 2, Section 4).

### Orthrus Model Overview

During the contrastive training phase, we sample positive pair sequences from mature RNA transcript sets and maximize their similarity in the model latent space (Figure 1 B). Given a batch of *N* reference sequences, $x_{1}^{1},\ldots ,x_{N}^{1}$, we construct positive pairs $\left(x_{i}^{1},x_{i}^{2}\right)$ by randomly sampling $x_{i}^{2}$ from the augmentation set of transcripts related to $x_{i}^{1}$ through alternative splicing or orthology processes, as described in the previous section. The augmentation set associated with each reference transcript can contain both splice isoforms and orthologous transcripts. The transcript positive pair from the augmentation set is re-sampled for each training epoch. We pass these positive pairs through a Mamba [31] encoder, *f_θ_* resulting in the outputs $h_{i}^{1}$ and $h_{i}^{2}$. These representations are then fed into a multi-layer perceptron projection head, *g_θ_* the output of which is used to calculate normalized projections, *z_i_*. We use the decoupled contrastive learning (DCL) loss [36] to perform the contrastive learning objective, pushing apart unpaired transcripts and maximizing the cosine similarity between positive pairs (Figure 1 B). We introduce two versions of Orthrus using a backbone Mamba encoder: Base consisting of 1.3 million trainable parameters and Large with 10.1 million trainable parameters (excluding *g_θ_*).

### Orthrus embeddings are predictive of diverse phenotypes

To evaluate the effectiveness of our pre-trained representations, we followed the conventional evaluation strategy of linear probing. The learned latent embedding is effective if ∃ **w** s.t. ${\text{w}}^{T}X+b=\hat{y}$, where, **X** is a matrix of embeddings and $\hat{y}$ approximates *y*. To evaluate the above, we freeze the weights of the Mamba encoder *f* and train a linear layer to predict labels for regression and classification tasks. Further experimental details are described in Appendix A.1.

**Fig. 2:**
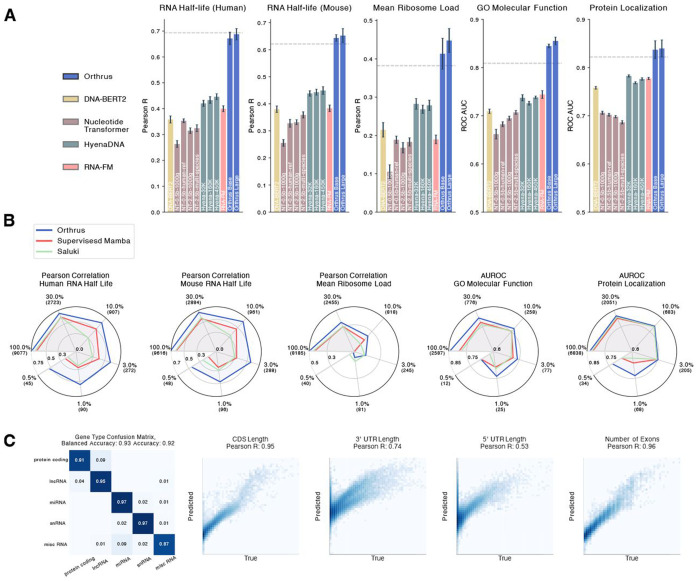
(**A**) Benchmarking linear probing performance on RNA property prediction tasks for self-supervised genomic foundation models. Individual bars represent the performance of foundation model variants, which typically differ in parameter count and pre-training dataset. Error bars show 95% confidence intervals, constructed using 10 runs with randomized data splits. The grey dashed line indicates the performance of the fully fine-tuned supervised Saluki method. (**B**) Plots evaluating the fine-tuning performance of Orthrus Base across varying data availability. Each dataset is subsampled to the indicated percentage, with the number of data points provided in brackets. Point estimates are plotted, averaged across three random seeds and random data splits. (**C**) Evaluation of Orthrus’s latent representation by fitting a linear model to predict structural properties. The confusion matrix evaluates Orthrus’s ability to classify transcript types using logistic regression on learned embeddings. The four scatter plots assess Orthrus’s ability to predict structural RNA properties, including CDS length, 3’ UTR length, 5’ UTR length, and number of exons.

We quantitatively evaluate whether Orthrus embeddings contain information regarding key biochemical properties such as UTR length, number of exons, CDS length, and gene type in Figure 2 C. We observe that Orthrus fixed length embeddings are highly predictive of RNA biochemical attributes, which are important for predicting functional RNA properties such as RNA half-life [7]. In Figure 2 A, we demonstrate that Orthrus outperforms other self-supervised methods on a diverse set of functional RNA property prediction tasks by a substantial margin. For RNA half-life (Human) Orthrus Large outperforms other self-supervised methods, the closest of which achieves 65% of linear probing performance (Pearson R 0.45 & 0.69). Further, we evaluate a Base 4 track model and find that Orthrus outperforms other self-supervised baselines (Table A1). We note that Orthrus outperforms a supervised baseline for most tasks, which is indicated by a dashed line (Figure 2 A). These results indicate that a linear regression trained with Orthrus embeddings can match or outperform neural networks tuned for RNA property prediction tasks [7].

We observe improved linear probing results as we scale the number of trainable parameters for Orthrus by comparing Base and Large model variants (Figure 2). We see an especially clear improvement trend in MRL and GO Molecular Function predictions. We note that for other self-supervised models such as Hyena DNA or Nucleotide Transformer, the number of parameters does not consistently improve performance (Figure 2 A) [14]. However, we do observe an improvement in performance for Nucleotide Transformer when comparing their 2.5 billion parameter model trained on 1000 genomes data versus multi-species [27]. This is additional evidence that utilizing evolutionary information can help improve model performance on RNA property prediction tasks [37].

### Fine-tuning Orthrus for state-of-the-art RNA property prediction

**Fig. A2:**
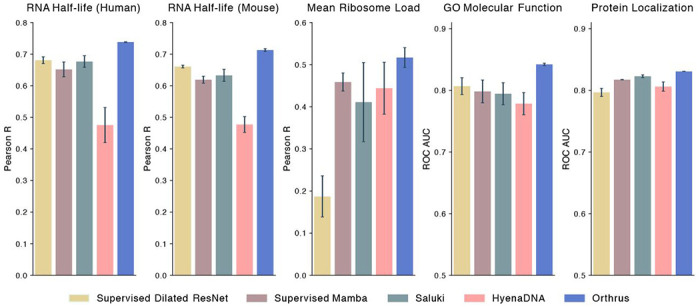
Fine tuning results plotted for 100% fraction of the data. Standard deviation is computed across 3 random seeds. Datasets are split with a homology aware strategy to avoid data leakage.

To assess whether the Orthrus pre-training objective provides utility beyond an effective representation, we evaluate its performance by fully fine-tuning it and comparing it to a supervised model with a matched architecture. We compare its performance against a published method for the RNA half-life prediction, Saluki [7], and find that the fully fine-tuned Orthrus model outperforms Saluki on the RNA half-life task (Figure 2). Furthermore, we retrain the Saluki architecture, train an architecturally equivalent model to Orthrus, and fine-tune pre-trained HeynaDNA model for other sequence property prediction tasks and identify that Orthrus has a significant performance advantage (Figures 2, A2). Other baseline SSL methods such as DNA-BERT2 and RNA-FM have limited input context windows, and cannot be easily applied to these tasks.

To simulate downstream tasks for which there is a lack of experimental data, we perform fine-tuning on RNA property prediction tasks where only a subset of the original training data set is available. We observe that supervised methods are ineffective in this regime, while Orthrus maintains competitive performance at 10% and 1% of the data (Figure 2 B). The performance differences are even more stark when using only 0.5% of the training data, achieving 73% of supervised performance with just 45 observed samples on the human RNA half-life dataset (R=0.72 & R=0.53). These findings illustrate that Orthrus advances towards the aim of few-shot learning for downstream tasks where experimental data is scarce.

### Orthrus latent space captures known functional transcript diversity

**Fig. 3:**
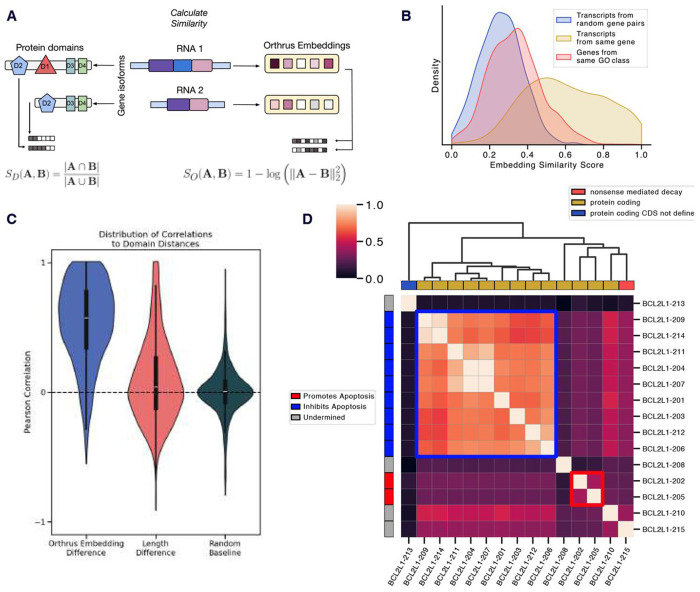
(**A**) Methodology for comparing gene isoform similarities using Orthrus embeddings and protein domain annotations. Orthrus embeddings for transcripts within the same gene are compared using log of L2 distance, while protein domain similarities are computed using the Jaccard index. (**B**) We visualize within gene similarities (yellow), between gene similarities (blue), and similarities of genes from the same GO class (red). (**C**) Visualization of Pearson R distributions correlating protein domain similarities with Orthrus embedding similarities for 1000 randomly sampled genes with multiple isoforms. Also plotted are the distributions of transcript length and protein domain similarities. (**D**) Example of BCL2L1 isoforms, where apoptosis-inhibiting isoforms cluster together, while non-coding and apoptosis-inducing isoforms display low similarity. The clustering matrix, derived from Orthrus embedding similarities, is represented by the dendrogram. Boundaries highlight clusters with divergent transcript functions.

A key question in alternative splicing research is how much functional diversity RNA isoforms generate within a gene [38]. To explore whether Orthrus embeddings can help elucidate this, we analyze intra-gene isoform similarities. For each pair of transcript isoforms within protein-coding genes, we compute their similarity using Orthrus embeddings (Figure 3 A). As a control, we compare these with transcript pairs from random genes, expecting lower similarity. We also hypothesized that transcript pairs from genes sharing the same GO terms would be more similar than random pairs, but less similar than most intra-gene pairs. Our analysis confirms significant differences across all pairwise comparisons of the three groups (p < 2.2e-16, Mann-Whitney U test), indicating that the Orthrus training objective preserves within gene sequence diversity (Figure 3 B). Notably, we observe an overlap between intra-gene and inter-gene similarities, indicating that some alternatively spliced transcripts have distinct embeddings, RNA properties, or functional differences in protein products. As such, *within gene* diversity could potentially help delineate differential isoform protein functions, an active area of research.

To investigate whether intra-gene similarities may reflect underlying protein domain conservation, we annotated each transcript, identifying a list of included protein domains. We found that transcripts with a high similarity, as measured by overlap in the present protein domains, also have highly similar Orthus embeddings. The correlation between Orthrus and domain similarities are significantly higher than a transcript length difference baseline, indicating that Orthrus learns functional differences as captured by domain presence (Figure 3 C). This suggests that Orthrus embeddings encode functionally relevant information.

To illustrate this, we examine the *BCL2L1* gene, known for its alternatively spliced isoforms with distinct functional outcomes [39, 40]. The dominant isoforms encode an apoptosis-inhibiting protein, Bcl-X(L), while a minority encode a pro-apoptotic protein, Bcl-X(S). By clustering *BCL2L1* RNA isoforms using Orthrus embedding similarity, we identify two main functional groups: one containing *BCL2L1-202* and *BCL2L1-205*, distinct from the apoptosis-inhibiting transcripts cluster (Figure 3 D). This demonstrates that Orthrus embeddings may serve as a valuable resource for identifying isoforms with likely different functional properties, a critical area in alternative splicing research.

### Ablations: Orthology and splicing saturate performance

Finally, we investigate the Orthrus augmentations that contribute towards effective performance. We find that both orthology and alternative splicing isoforms are able to achieve high performance. In addition, the six track representation is effective for RNA property prediction tasks that are known to be influenced by exon junction density such as RNA half-life prediction [41]. Introduction of masking improves all around performance, which could be due to preventing shortcut learning [15, 42].

## 3 Discussion

As Dobzhansky famously notes: “Nothing in biology makes sense except in the light of evolution” [43]. Orthrus similarly aims to capture the diversity of RNA through an evolutionary and functional lens [44, 45]. We create a self-supervised training objective that learns similarities between evolutionarily related sequences identified in the Zoonomia project [30]. In addition, we utilize alternatively spliced transcripts to learn sequences responsible for shared functions between splicing isoforms [46]. By training on sequences generated by evolutionary and alternative splicing processes, Orthrus utilizes stronger biologically motivated inductive biases compared to SSL reconstruction methods. This makes Orthrus less reliant on limited genetic sequence diversity during pre-training, and capable of learning strong representations without fine-tuning on experimental data.

Previous self-supervised works for genomic sequence property prediction have focused on reconstruction objectives like masked language modeling or next token prediction [12, 27]. However, most positions in the human genome are under little to no negative selection, and are not as informative for model training [23, 24]. Thus, predicting the corresponding tokens introduces little new information to the model.

We demonstrate that by minimizing the distance between mature RNAs generated through speciation and alternative splicing, we are able to generate representations useful for RNA property prediction tasks. We empirically demonstrate that Orthrus embeddings contain information useful for predicting RNA properties like RNA half-life and mean ribosome load, and achieves state-of-the-art prediction when fine-tuned. We observe that pre-training is especially helpful in low data regimes when there are 200 or fewer data points with labels. We demonstrate that self-supervised pre-training is an approach for addressing data efficiency challenges present in genomics, and scaling to additional species can be an effective dataset expansion strategy.

An important question to address is why we expect that minimizing distances between RNA isoforms would be useful for predicting phenotypes like RNA half-life or protein localization. One hypothesis is that alternative splicing and speciation events preserve core functional RNA segments. Through the contrastive pre-training procedure, we identify these shared regions between diverse sequences. Indeed, a recent work proposes that contrastive methods are effective due to block separating latent variables shared between augmented samples [47]. This view is supported by our findings identifying that Orthrus within gene similarities are correlated with domain presence. By utilizing decoupled contrastive learning, diverse sequences are pushed apart, thus uniformly distributing samples in the latent space, which helps with downstream task performance [36, 48]. Through encoding these invariances, we find that Orthrus is able to learn complex RNA properties such as cellular component localization and RNA half-life.

A possible limitation of our approach is that by maximizing similarity in representation space between functionally related sequences, we remove important signals for predicting properties. Are there property prediction tasks for which our inductive bias is actually detrimental compared to a randomly initialized model? For RNA half-life, [49] demonstrated that in more than 85% of genes, isoform choice has no statistically discernible effect. Indeed, we find that within gene Orthrus embeddings still demonstrate significant diversity, demonstrating capability of reflecting within gene variable function. There are other processes for which it is widely considered that alternative splicing is functionally crucial, such as the development of neurological tissues [50, 51].

In this work, we propose a novel, self-supervised contrastive objective for learning mature RNA isoform representations. We show that this approach is an effective strategy to address two major challenges for cellular property prediction: data efficiency, and model generalizability. We demonstrate that Orthrus representations are effective in the low data setting, paving the path to true few-shot learning for RNA property prediction. Finally, we outperform supervised models when fine-tuning Orthrus and significantly improving over performance of reconstruction based self-supervised methods. These findings open the possibility that combining the contrastive loss with a masked language modelling objective can further improve quality of mature RNA representations.

## 4 Methods

Contrastive learning has been shown to be a bound on mutual information between two random variables X and Y corresponding to $I\left(X;Y\right)=E_{p\left(x,y\right)}\left[\log\frac{p\left(x,y\right)}{p\left(x\right)p\left(y\right)}\right]$. We utilize a variation of the classical InfoNCE loss, $E\;\left[\log\frac{\exp\left(f\left(x_{i},y_{i}\right)\right)}{\sum \exp\left(f\left(x_{i},y_{i}\right)\right)}\right]$, where a model *f* is tasked with classifying the correct *y*_*i*_ which was jointly drawn with *x_i_* [52]. Herein, the observations *x_i_*, *y_i_* correspond to splice isoforms or orthologous sequences which are interpreted as functionally related while *f* is a neural network that we optimize to minimize the loss.

We propose to use 4 different augmentations and thoroughly investigate their impact on downstream tasks. They include: alternatively spliced transcripts across ten species, orthologous transcripts identified from the Zoonomia project including over 400 species, naive orthology informed by gene identity, and masking a percentage of the input sequence (Figure 1) [30, 33].

In the following section we elaborate on dataset construction, model choice, contrastive learning objective, and downstream evaluations.

### Splicing and Orthology Contrastive Dataset

In the vision domain, contrastive learning strategies have had significant success by identifying augmentations that do not have a strong semantic effect, such as cropping, rotation, or Gaussian blur [29, 53, 54]. In this work, we use RNA splicing isoforms and orthologous transcripts as sources of functional similarity [30, 32, 33]. By sampling RNA isoform sequences produced by alternative splicing and speciation processes, we identify sequence variation that is likely to maintain core functional properties. In addition, we use naive orthology to pool RNA transcripts from evolutionarily related genes [46]. By minimizing the distance between functionally similar sequences, the model can learn regulatory regions critical for RNA property and function prediction.

For mRNA sequence representation we generate a six-track mature RNA representation, consisting of four one-hot encoded tracks encoding genomic sequence, a track indicating the 5’ location of splice sites, and a track indicating the first nucleotide of every codon. The addition of splice site and coding sequence locations has been shown to be beneficial for mRNA property prediction tasks [7].

To sample positive pairs from the orthology and splicing dataset, we first identify the set of all positive samples **Y**_j_ for a reference transcript *x_j_*. **Y**_j_ can be variable in length since some transcripts will have a greater number of splice isoforms and orthologous sequences than others. During a forward model pass, we sample *$y_{j}^{k}$* from **Y**_j_ and use that as a positive pair for *x_j_*.

### Mamba Encoder

We pre-train a Mamba state space model, which has been demonstrated to be successful in applications with long context requirements [14, 31]. mRNA sequences can reach over 12,000 nucleotides in length, making application of the Transformer architecture challenging due to its quadratic scaling in memory with sequence length [55]. Mamba, an extension of state space model families or S4, maps a sequence x(t)∈ℝ to y(t)∈ℝ using a latent state $h\left(t\right)\in {\mathbb{R}}^{N}$ [56].

A fundamental trade-off in architecture choice for sequence modeling is avoiding compressing sequence context and compute requirements. Transformers are able to avoid compressing context, leading to better performance, but trade-off slower training and higher memory usage [31, 55]. Alternatively, S4 models define a sequence to sequence transformation parameterized by (**A**, **B**, **C**, Δ). The fundamental operation consists of iteratively updating the hidden state:

h′(t)=Ah(t)+Bx(t)y(t)=Ch(t).

Δ is used to discretize the input for discrete domains such as natural language, or genomics. The Mamba architecture iterates on the S4 family of models by introducing selectivity over input by making *B*, *C*, and Δ a function of the input, resulting in

$$h\prime \left(t\right)=\text{A}h_{t}+\text{B}\left({\text{x}}_{\text{t}}\right)x_{t}\;\;\;\;y\left(t\right)=\text{C}\left({\text{x}}_{\text{t}}\right)h_{t}.$$

Allowing parameters to be input dependent introduces desirable modeling qualities for genomic domain: variable spacing, filtering context, and linear memory scaling with sequence length 𝒪(n). Variable spacing refers to Mamba’s ability to effectively perform on the selective copying task, where causal elements are arbitrarily spaced [31]. Binding motifs in genomic sequences can be spaced without a constant offset, requiring the model to be able to learn motif interactions with variable spacing [57]. The non-unformity of signal informativeness in genomic sequences requires models to be able to filter out irrelevant context [31]. Finally, the limited context, as opposed to Transformer models, allows the Mamba architecture to scale required memory linearly with increased input length [31, 55].

### DCL Contrastive Learning Objective

We use decoupled contrastive learning (DCL) as it has been shown to require smaller batch sizes, is less sensitive to hyperparameters such as learning rate, and the positive loss term can be weighted by sample difficulty [36]. DCL iterates on the normalized temperature-scaled cross-entropy loss by splitting the contrastive objective into two terms: a similarity loss (positive) and a dissimilarity loss (negative) [58]. More formally, the positive and negative losses for sample *i* are calculated:

(1) $${ℒ}_{DCL,i}\left(\theta \right)=\log\;\left[\sum_{k=1}^{N},\sum_{l=1}^{2}1_{k\neq i}\exp\left(\langle z_{i}^{1}\cdot z_{k}^{l}\rangle /\tau \right)\right]-w_{i}\langle z_{i}^{1}\cdot z_{k}^{l}\rangle /\tau .$$

In the above *z*^1^ and *z*^2^ correspond to two embeddings of related sequences, *z_k_* are embeddings from unrelated RNA sequences, τ is the temperature parameter set to 0.1, and $1_{k\neq i}$ is an indicator function that evaluates to 1 when k≠i. The above loss is computed for all the samples in the batch for both the sampled views *l* ∈ 1, 2. *N* corresponds to all the negative samples in batch, thus maximizing batch size during contrastive learning typically leads to improved performance.

Normalized projections *z_i_* are outputs from the MLP projector *g_θ_* and are used to compute the contrastive loss, utilizing samples from the rest of the batch as negative examples:

(2) $$z_{i}^{1}=\frac{g\left(h_{i}^{1}\right)}{\left\|g\left(h_{i}^{1}\right)\right\|}\;\text{and}\;z_{i}^{2}=\frac{g\left(h_{i}^{2}\right)}{\left\|g\left(h_{i}^{2}\right)\right\|}.$$

For downstream RNA property evaluations, the projector *g_θ_* is discarded and outputs from *f_θ_* are used instead. This practice is consistent with prior literature [29, 59–61].

### Downstream Evaluation Tasks

**RNA half-life** (RNA HL) is an important cellular property to measure due to its implications for protein expression regulation. Recently, it has been shown that the choice of experimental methodology for measuring RNA half-life can have an outsized impact [7]. To address this challenge, Agarwal and Kelley (2022) utilized the first principal component of over 40 different RNA half-life experiments. The dataset consists of 10,432 human and 11,008 mouse RNA sequences with corresponding measurements. The low data availability and high inter-experiment variation underscore the importance of data efficiency, and generalizability in computational models to be developed for this task.

**Mean ribosome load (MRL)** is a measure of the translational efficiency of a given mRNA molecule. It measures the number of ribosomes translating a single mRNA molecule at a point in time. Accurate MRL measurement is crucial as it offers insights into the efficiency of protein translation, a key process in cellular function. The dataset in question, derived from the HP5 workflow, captures this metric across 12,459 mRNA isoforms from 7,815 genes [62]. This dataset was derived from a single experiment, so we can expect a higher amount of noise associated than the RNA half-life dataset.

**Protein localization** Protein function is often linked to its subcellular location, which can be determined using cells that are immunofluorescently stained. We downloaded a dataset of 10,409 genes, whose protein localization was determined by the Human Protein Atlas [63]. We included the 12 most common locations including Nucleoplasm, Cytosol, Vesicles, Mitochondria, Plasma Membrane, Golgi apparatus and others. We utilized one transcript per gene (defined to be the canonical isoform by Appris database [64]).

**Gene ontology** (GO) terms are a hierarchical classification system used for assigning function to genes and their products [65–67]. In this work, we utilize GO classes to visualize model latent embeddings and classification. GO term hierarchical systems allow for fine-grained annotation of function, with broader terms at the top of the hierarchy and increased specificity closer to the bottom. To annotate genes with gene ontology terms, we subset GO classes three levels from the root, labeling all available genes.

#### 4.1 Associating Orthrus RNA Embeddings with Transcript Similarity and Protein Domains

To evaluate how well Orthrus RNA embeddings capture functional diversity among transcript isoforms, we analyzed the similarity of transcript pairs within and between protein-coding genes, excluding homologous genes when comparing random gene pairs or genes sharing the same GO term. The test dataset for this analysis was prepared as follows:

1. **Intra-gene Pairs:** We sampled 1,000 genes to obtain pairs of protein-coding transcripts.
2. **Inter-gene Pairs:** We randomly sampled 1,000 pairs of non-homologous genes, selecting the MANE transcript for each gene, which represents the most likely relevant isoform.
3. **Inter-gene Pairs:** We sampled 5,000 GO terms, each containing 10 to 1,000 genes, and selected five non-homologous gene pairs per term.

For each transcript, we computed Orthrus embeddings and calculated pairwise distances between embeddings using the L2 norm. We calculated a similarity score for each transcript pair as 1 − log(L2 distance). This ensures more interpretable results, where higher similarity scores correspond to closer RNA embeddings in the latent space, allowing us to compare the three groups of transcript pairs.

To assess whether similarities in Orthrus embedding reflected shared functional features, we annotated each transcript with protein domain information using Ensembl data and the Pybiomart package. We used the Jaccard Index to quantify the similarity of protein domain presence or absence between each pair of transcripts within a gene. The Jaccard Index is defined as the size of the intersection divided by the size of the union of the protein domain sets present in each transcript pair:

$$\text{Jaccard Index}=\frac{\left|D_{1}\cap D_{2}\right|}{\left|D_{1}\cup D_{2}\right|}$$

where *D*_1_ and *D*_2_ are the sets of protein domains present in each transcript. Higher values indicate greater similarity in protein domain composition. We calculated this metric using ”intra-gene pairs” and ”inter-gene pairs” to further study how protein domain composition correlated with embedding similarity. We analyzed the Pearson correlation between Jaccard indices and embedding similarities separately for intra-gene and inter-gene pairs to determine if transcript pairs within the same gene exhibited higher concordance.

To further explore the utility of Orthrus embeddings, we conducted a detailed analysis of the *BCL2L1* gene [39]. Transcripts from this gene were clustered based on their Orthrus embedding similarity scores, with clusters visualized and annotated according to transcript type and known functional roles.

## Figures and Tables

**Table 1:** Ablation results are generated using linear probing, by fitting a linear model on pre-computed embeddings from Orthrus base models. 6t; Six track input corresponding to one hot encoded sequence, splicing and codon positions. Masking corresponds to randomly masking 15% of the input sequence.

Splice	Orthology	6 tracks	Masking	RNA HL Human R	RNA HL Mouse R	MRL R	GO MF ROC AUC	Protein Loc. ROC AUC
**✓**	**✓**	**✓**	**✓**	0.675	0.615	0.393	0.845	0.834
**✗**	**✓**	**✓**	**✓**	0.678	0.610	0.392	0.842	0.833
**✓**	**✗**	**✓**	**✓**	0.680	0.615	0.402	0.854	0.834
**✓**	**✓**	**✗**	**✓**	0.531	0.512	0.361	0.833	0.825
**✓**	**✓**	**✓**	**✗**	0.647	0.595	0.332	0.836	0.822
**✗**	**✗**	**✗**	**✗**	0.217	0.214	0.114	0.753	0.792

**Table 2:** Overview of contrastive datasets.

Contrastive Dataset	Zoonomia	Splicing	# of Pairs	# of Transcripts
Zoonomia Eutheria & Splicing Gencode Basic	**✓**	**✓**	876,871,640	49,493,993
Zoonomia Eutheria	**✓**	**✗**	157,975,815	41,562,358
Splicing Gencode Basic	**✗**	**✓**	16,249,112	771,105
None	**✗**	**✗**	0	771,105

**Table 3:** Overview of evaluation datasets. Locality refers to whether the model is required to reason over global structure or be able to pick up local signals.

Task Dataset	Category	Locality	Number of Sequences	Maximum Sequence Length	Homology Split Possible	Species
RNA Half Life Human	Regression	Global	12968	12288	**✓**	Human
RNA Half Life Mouse	Regression	Global	13738	12288	**✓**	Mouse
Mean Ribosome Load	Regression	Global	11693	12275	**✓**	Human
Protein Localization	Classification	Global	9769	12275	**✓**	Human
Gene Ontology MF	Classification	Global	3697	12236	**✓**	Human

**Fig. A1:**
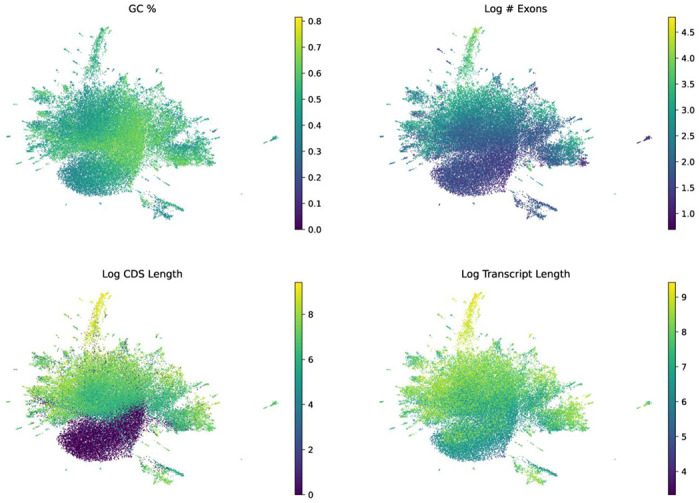
UMAP visualization of the Orthrus Large embeddings. 50,000 transcripts are randomly sampled from gencode comprehensive and colored according to transcript length, number of exons, CDS length, and GC%.

## Footnotes

1 In the coding region (2% of human DNA), an average individual carries 27 ± 13 unique SNPs [21].

## Appendix A

### A.1 Linear Probe Experimental Details

In this section, we describe the experimental procedure to evaluate linear probing results.

We first performed a 70-15-15 data split on datasets. The data sequences are then embedded by the various self-supervised learning (SSL) models. For Orthrus, we simply take the mean of the embeddings across the seqeunce dimension. For HyenaDNA, we take the mean and max of the embedding sequence dimension, as well as the last hidden state in the output sequence. Other SSL methods could not handle input sequences of more than 500 or 1000 nucleotides. Thus, when input sequences exceeded the allowable context window, each sequence was chunked to the maximum length allowed by a model. We then computed the mean of each chunk embedding across the sequence dimension, and then averaged the mean embedding of each chunk to obtain the final embedding.

After obtaining embedding vectors, we used the scikit-learn implementation of linear models to perform the linear probes of the embeddings. For the downstream regression tasks, we used either used linear regression or ridge regression with the regularization parameter selected by cross validation. The final linear model was selected using the validation split. The gene ontology and protein localization tasks are multi-label classification tasks. For this, we fit scikit-learn’s LogisticRegression model to the labels using a MultiOutputClassifer, which essentially trains a separate linear classifier for each label class. We use the default logistic regression parameters, and set 5000 maximum iterations for the solver. Below is the table with the performance numbers that are reported in Figure 2

**Table A1:** Linear probing results for self-supervised methods. The embeddings were computed for each method and then linear regression was computed analytically using the corresponding labels for each task. Bolded numbers indicate the best performing model.

Model	RNA HL Human	RNA HL Mouse	MRL R	GO MF ROC AUC	Protein Loc. ROC AUC
DNA-BERT2	0.36 ± 7e-3	0.38 ± 6e-3	0.21 ± 9e-3	0.71 ± 2e-3	0.76 ± 1e-3
NT-500m-1000g	0.26 ± 7e-3	0.26 ± 6e-3	0.11 ± 9e-3	0.66 ± 5e-3	0.71 ± 2e-3
NT-500m-human-ref	0.35 ± 4e-3	0.33 ± 7e-3	0.19 ± 5e-3	0.68 ± 3e-3	0.70 ± 1e-3
NT-2.5b-1000g	0.31 ± 5e-3	0.33 ± 4e-3	0.17 ± 7e-3	0.69 ± 2e-3	0.70 ± 1e-3
NT-2.5b-multi-species	0.32 ± 7e-3	0.36 ± 6e-3	0.18 ± 6e-3	0.71 ± 2e-3	0.69 ± 2e-3
Hyena-32K-seqlen	0.42 ± 6e-3	0.44 ± 5e-3	0.28 ± 7e-3	0.74 ± 3e-3	0.78 ± 1e-3
Hyena-160K-seqlen	0.43 ± 7e-3	0.44 ± 6e-3	0.27 ± 5e-3	0.73 ± 2e-3	0.77 ± 1e-3
Hyena-450K-seqlen	0.45 ± 6e-3	0.45 ± 7e-3	0.28 ± 7e-3	0.74 ± 1e-3	0.78 ± 1e-3
RNA-FM	0.40 ± 6e-3	0.38 ± 6e-3	0.19 ± 6e-3	0.74 ± 4e-3	0.78 ± 1e-3

Orthrus Base 4 track	0.52 ± 2e-2	0.54 ± 2e-2	0.38 ± 5e-3	0.84 ± 6e-3	0.83 ± 9e-3
Orthrus Base	0.67 ± 1e-2	0.64 ± 6e-3	0.41 ± 2e-2	0.84 ± 2e-3	0.84 ± 9e-3
Orthrus Large	**0.69** ± 1e-2	**0.65** ± 1e-2	**0.45** ± 2e-2	**0.86** ± 4e-3	**0.84** ± 9e-3

To avoid information leakage we perform homology splitting within each species. Additional details are described in A.2.

In addition, to further evaluate the quality of the model latent space we visualized the learned embeddings by performing dimensionality reduction using UMAP (Figure A1) [68]. Each point in the figure corresponds to a transcript and is colored depending on the biochemical property of interest. We observe clear separation of transcripts conditioned on the GC % CDS length, number of exons, and transcript length. This ensures that information regarding important biochemical properties is preserved in the model embeddings even after the length pooling operation.

### A.2 Homology splitting

To perform homology splitting we first acquire paralog information from Ensembl for a species of interest [69]. Ensembl provides pairs paralog information in the form of gene pairs related through duplication events. However, to perform homology splitting between genes we want to make sure that paralog transitivity is taken into account when dividing training samples between train, validation, and test splits. For example given three genes *g*_1_, *g*_2_, *g*_3_ if *g*_1_, *g*_2_ are annotated as paralogs and *g*_2_, *g*_3_ are annotated as well we want to ensure that *g*_1_, *g*_3_ are in the same split. Thus, we first transform the pairwise relationships into a graph structure in the process pruning low confidence paralog relationships. We enforce a similarity threshold of 35% which empirically demonstrated highly connected groups of paralogs. The algorithm for grouping is described in [27].

Following construction of the homology graph, during train-val-test split samples associated with genes which are connected in the homology graph are indexed into the same split. This avoids information leakage due to homology relationships within a given species.

### A.3 Fine-tuning Experimental Details

We fine-tune Orthrus by first initializing most of the model with weights from pre-training, the penultimate two layers with random initialization, and the final layer with zero initialization. We don’t apply any weight decay to weights that were initialized from pre-training while the final three layers have an l2 weight decay term of 1e-5. We fine-tune on downstream tasks using the Adam optimizer with a learning rate of 0.01. We apply exponential learning rate decay with a factor of 0.95. The models are trained with a single Nvidia T4 GPU in a mixed precision setting.

HyenaDNA models initialized with fine-tuning head. We perform a small learning rate hyperparameter grid search around the suggested hyperparameters of 6e-4. The suggested AdamW optimizer is used. Models were trained for a maximum of 100 epochs on Nvidia T4 GPUs with a batch size of 28 for the HyenaDNA-tiny and a batch size of 8 for HyenaDNA-small. Models were stopped early based on validation loss using an epoch patience of three. After selecting learning rate using the validation split, the runs were repeated using different random initializations to generate confidence intervals.

**Algorithm 1:** Homology Group Assignment

**Input:** Set of genes *G*, Set of paralogous relationships *P*, Similarity threshold *s*
**Output:** Homology map assigning genes to groups
**Initialize:**
*group_counter* ← *0*
*homology_map* ← {}
**Filter:**
*P* ← {(*g*1, *g*2) € *P* | similarity(*g*1, *g*2) > *s*}
**for each** (*g*1, *g*2) **in** *P* **do**
**if** *g*1 ∉ *homology_map* **and** *g*2 ∉ *homology_map* **then**
*homology_map*[*g*1] ← *group_counter*
*homology_map*[*g*2] ← *group_counter*
*group_counter* ← *group_counter* + 1
**else if** *g*1 ∉ *homology_map* **and** *g*2 ∉ *homology_map* **then**
*homology_map*[*g*2] ← *homology_map*[*g*1]
**else if** *g*2 ∉ *homology_map* **and** *g*1 ∉ *homology_map* **then**
*homology_map*[*g*1] ← *homology_map*[*g*2]
**else if** *homology_map*[*g*1] ≠ *homology_map*[*g*2] **then**
*old_group* ← *homology_map*[*g*2]
*new_group* ← *homology_map*[*g*1]
**for each** gene **in** homology_map **do**
**if** *homology_map*[*gene*] == *old_group* **then**
*homology_map*[*gene*] ← *new_group*
**end if**
**end for**
**end if**
**end for**
**Return** *homology_map*

**Table A2:** Pearson correlations (R) and ROC AUC of full model fine-tuning on RNA half-life (HL), mean ribosome load (MRL), gene ontology molecular function (GO MF) classification, protein localization and mRFP expression tasks. Best models are shown in bold. Performance values were averaged over 3 random seeds. Training validation and test were split based on sequence homology to prevent data leakage.

Models	RNA HL Human R	RNA HL Mouse R	MRL R	GO MF ROC AUC	Protein Loc. ROC AUC	Number of Parameters	Number of Tracks
Mamba Base Supervised	0.65 ± 2e-2	0.62 ± 1e-2	0.46 ± 2e-2	0.8 ± 2e-2	0.82 ± 3e-4	1.3m	6
Dilated CNN Resnet	0.68 ± 9e-3	0.66 ± 4e-3	0.13 ± 1e-1	0.81 ± 1e-2	0.8 ± 6e-3	0.83m	6
Saluki	0.68 ± 2e-2	0.63 ± 2e-2	0.41 ± 8e-2	0.79 ± 2e-2	0.82 ± 2e-3	0.15 m	6
HeynaDNA Small	0.48 ± 5e-2	0.48 ± 2e-2	0.44 ± 5e-2	0.78 ± 2e-2	0.81 ± 7e-3	3.3m	4
Orthrus Base	**0.74** ± 9e-4	**0.71** ± 4e-3	**0.52** ± 2e-2	**0.84** ± 2e-3	**0.83** ± 6e-5	1.3 m	6
